# Supplementary material for: A CD26+ tendon stem progenitor cell population contributes to tendon repair and heterotopic ossification
Source: Nat Commun. 2025 Jan 16;16:749. doi: 10.1038/s41467-025-56112-5 (PMC11739514; doi:10.1038/s41467-025-56112-5)
Supplement: Supplementary file 1 — Supplementary information [file 41467_2025_56112_MOESM1_ESM.pdf]

Supplementary figures

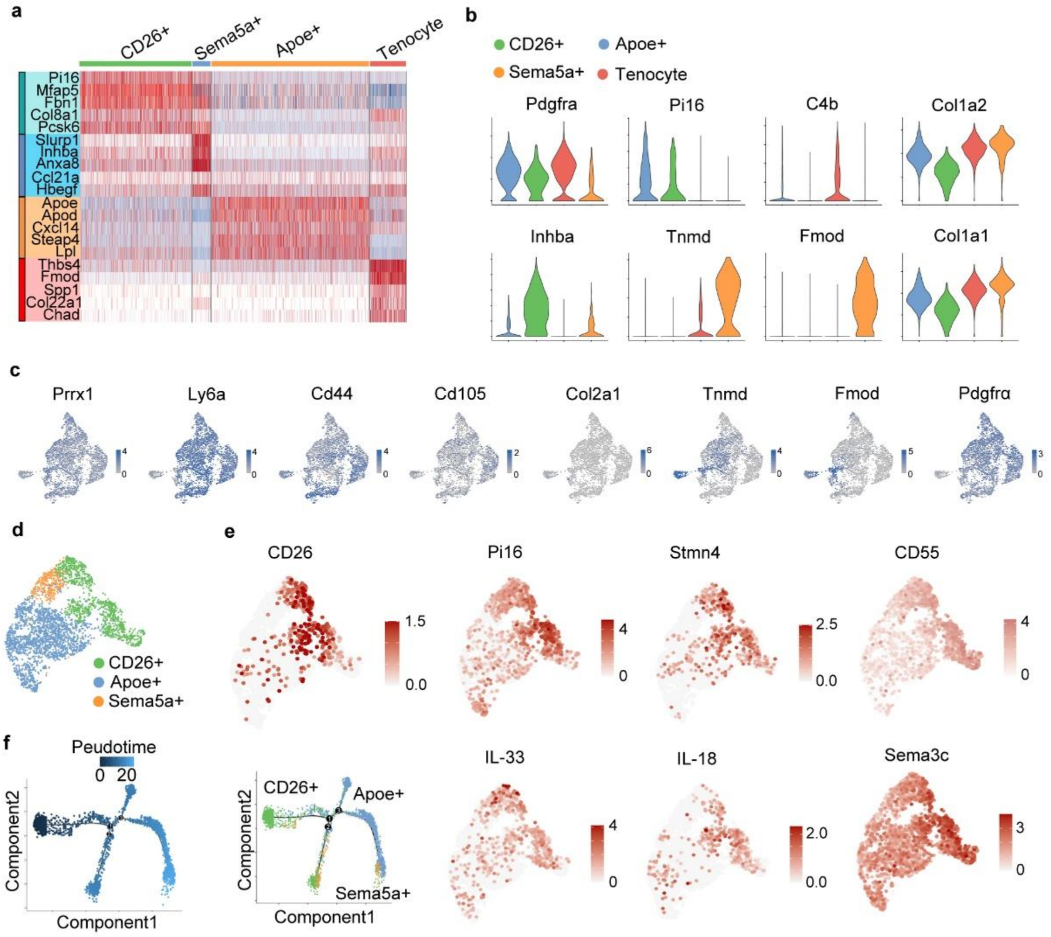

Figure S1. A, Heat map of key genes in CD26^{+} MSCs, Apoe^{+} progenitors, Sema5a^{+} progenitors and tenocytes. B, Violin plot of key genes of CD26^{+} MSCs, Apoe^{+} progenitors, Sema5a^{+} progenitors. C, UMAP plot of mesenchymal stromal cell, chondrocyte and tenocyte marker genes in CD26^{+} MSCs, Apoe^{+} progenitors, Sema5a^{+} progenitors. D, UMAP plot of CD26^{+} MSCs, Apoe^{+} progenitors, Sema5a^{+} progenitors and tenocytes. E, UMAP plot of key genes in CD26^{+} MSCs. F, Peudotime analysis of CD26^{+} MSCs, Apoe^{+} progenitors and Sema5a^{+} progenitors with Monocle.

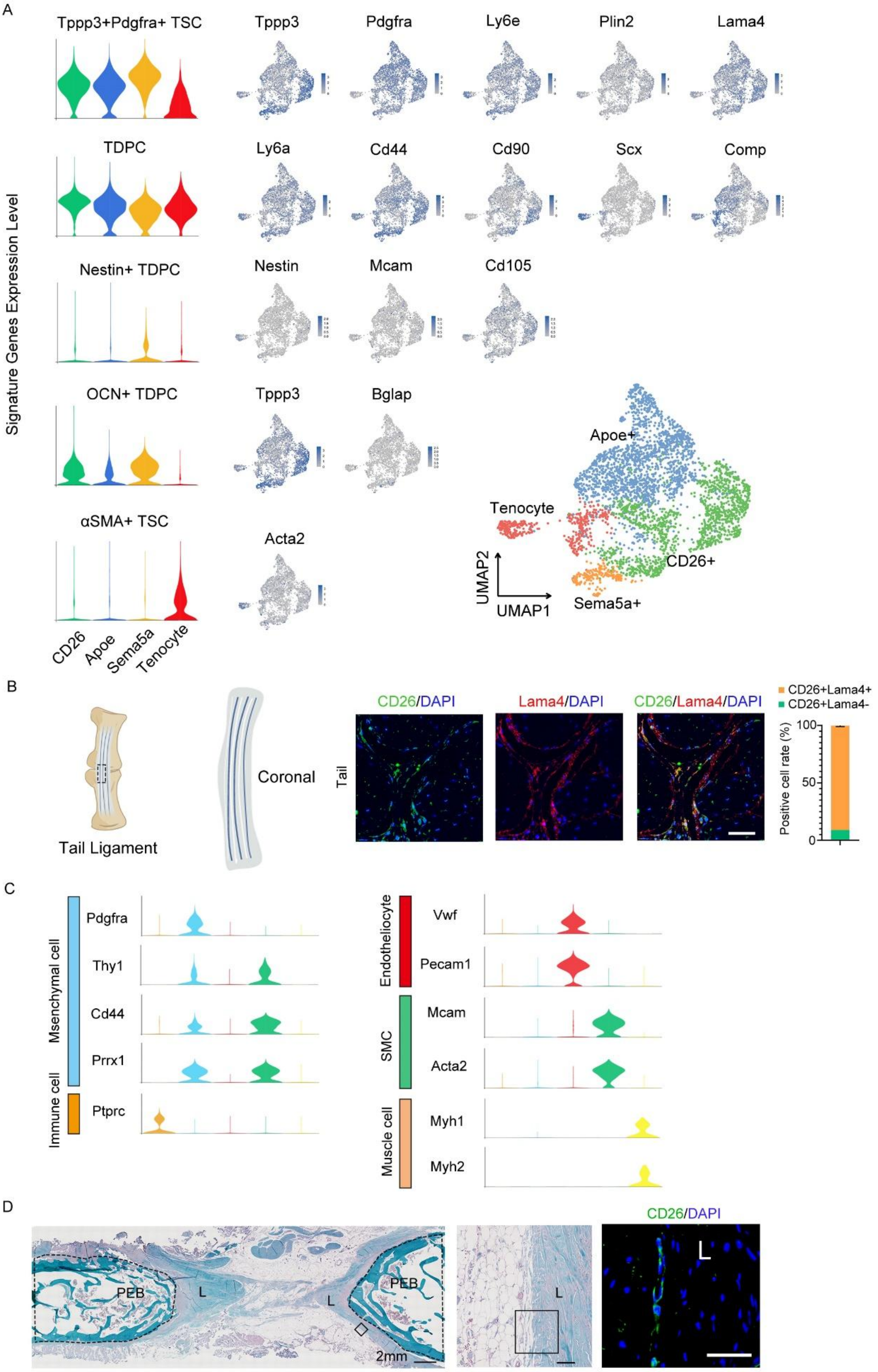
A
Tppp3+Pdgfra+ TSC
Tppp3
Pdgfra
Ly6e
Plin2
Lama4
TDPC
Ly6a
Cd44
Cd90
Scx
Comp
Nestin+ TDPC
Nestin
Mcam
Cd105
OCN+ TDPC
Tppp3
Bglap
αSMA+ TSC
Acta2
Signature Genes Expression Level
CD26
Apoe
Sema5a
Tenocyte
Apoe+
Tenocyte
CD26+
Sema5a+
UMAP2
UMAP1
B
Tail Ligament
Coronal
Tail
CD26/DAPI
Lama4/DAPI
CD26/Lama4/DAPI
CD26+Lama4+
CD26+Lama4-
Positive cell rate (%)
100
50
0
C
Msenchymal cell
Pdgfra
Thy1
Cd44
Prrx1
Immune cell
Ptprc
Endotheliocyte
Vwf
Pecam1
SMC
Mcam
Acta2
Muscle cell
Myh1
Myh2
D
PEB
L
L
PEB
2mm
L
CD26/DAPI
L

Figure S2. A, Violin plot and UMAP plot of signature genes of previously reported tendon stem/progenitor cells in $CD26^{+}$ MSCs, $Apoe^{+}$ progenitors, $Sema5a^{+}$ progenitors and tenocytes. B, Left, scheme for murine tail tendon collection. Right, immunofluorescent analysis and quantification of CD26 and Lama4 in cross section of tail tendon from adult mice. n=3. Scale bar: 100μm. C, Violin plot of expression of key genes in each cluster. D, SOFG staining and immunofluorescent analysis of CD26 in human spinal ligaments from n=3 samples. Scale bar: 2mm (left), 100μm (middle and right). SP, spinal process; IL, interspinal ligament; SL, supraspinal ligament; PEB, peri-entheseal bone; L, ligaments.

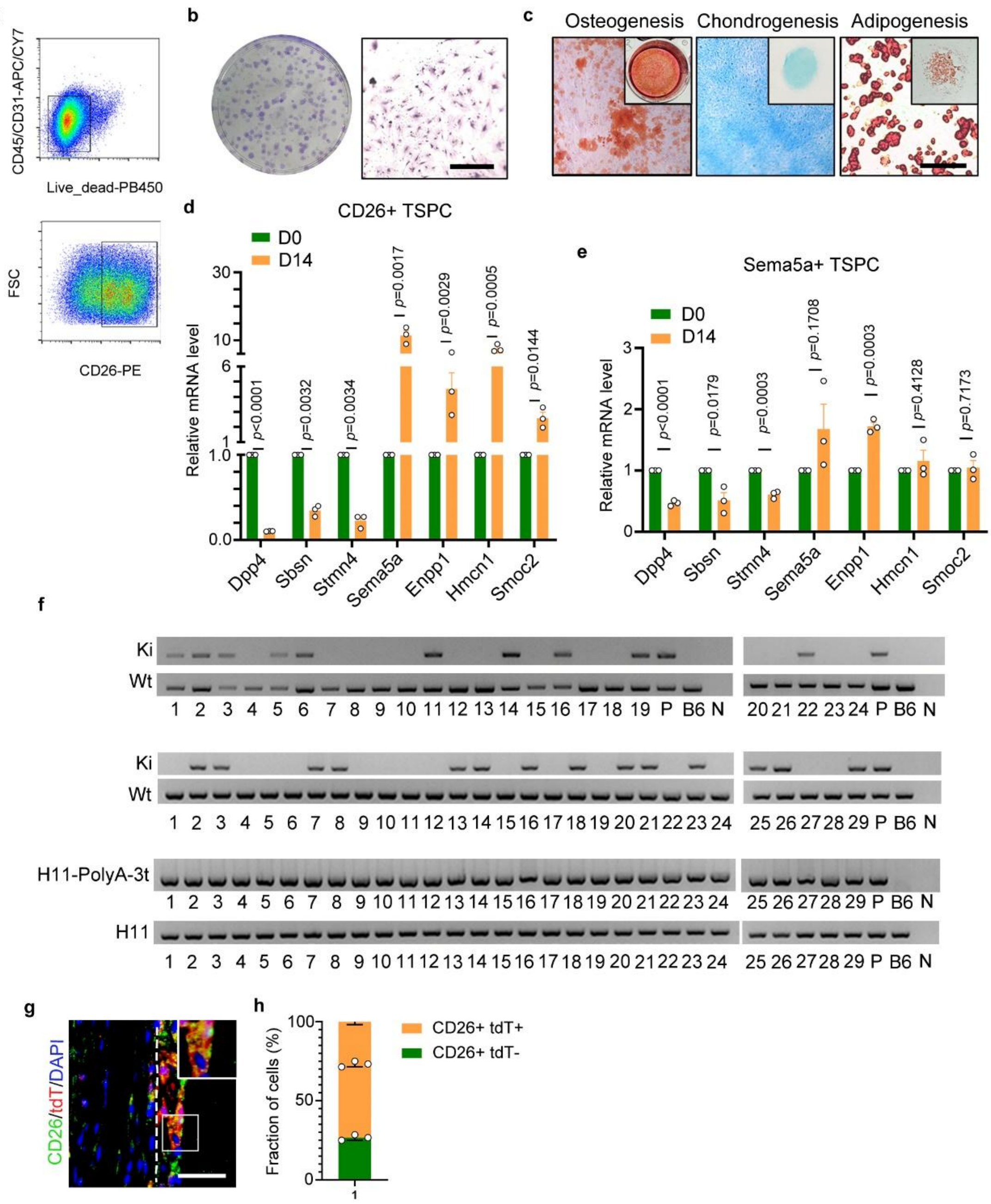

Figure S3. A, Fluorescence activated cell sorting (FACS) strategy of CD45$^-$CD31$^-$CD26$^+$ from human tendon. B, Proliferation assay of CD45$^-$CD31$^-$CD26$^+$ cells from human tendon. n=3. C, Alizarin red, alcian blue and oil red O staining of CD45$^-$CD31$^-$CD26$^+$ cells. n=3. Scale bar: 100μm. D-E, qPCR analysis of signature genes of CD26$^+$ TSPC and Sema5a$^+$ progenitor. n=3. Two-tailed unpaired Student's t test. F, Identification of *CD26*-CreER^{T2}-hDTR mice and *CD26*- CreER^{T2}-hDTR; H11-mZsGmtdT mice. G-H, Immunofluorescent staining and quantification of CD26 and tdT after tamoxifen induction in *CD26*-CreER^{T2}-hDTR mice. Data shown are mean ± SEM. Source data are provided as a Source Data file.

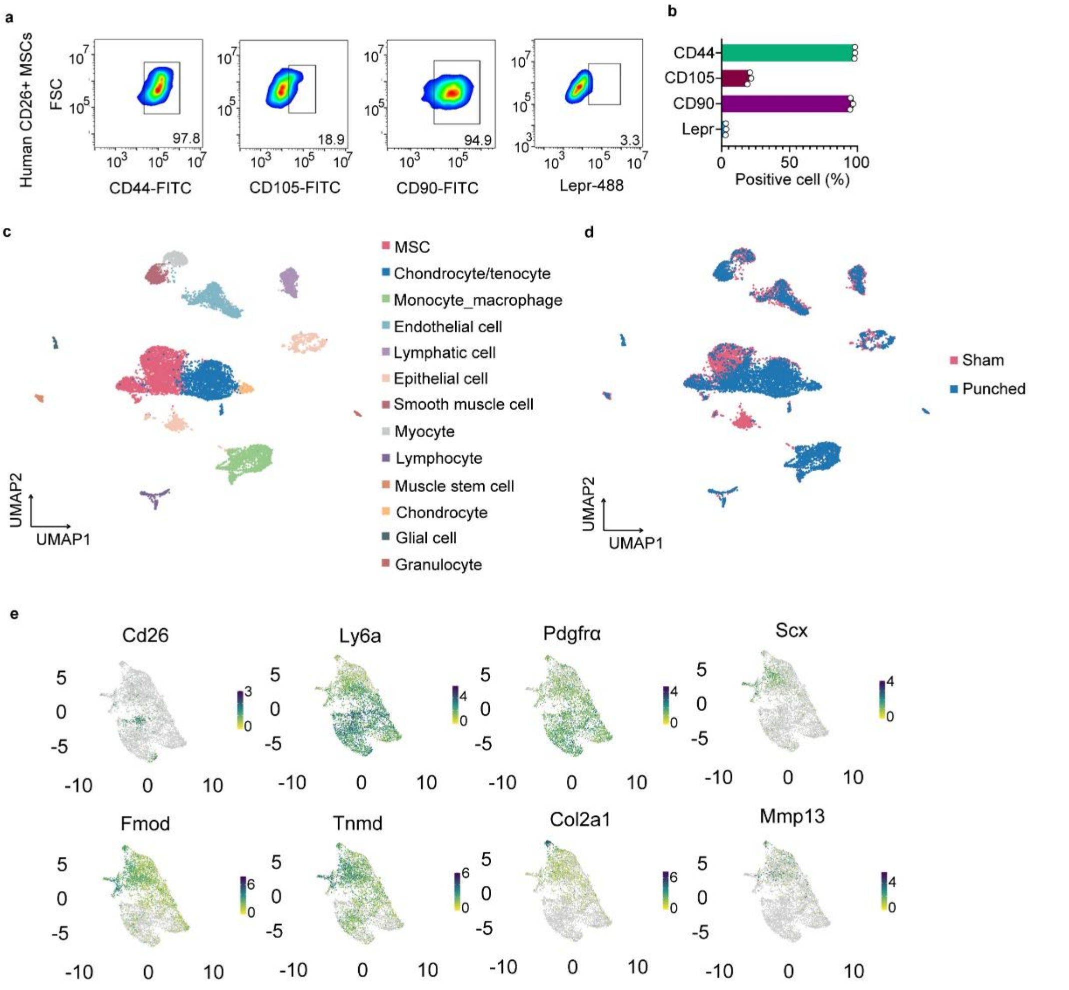

Figure S4. A-B, Flow cytometry analysis of stem cell markers from human ligaments (CD44, CD105, CD90 and LepR) in CD26$^+$ cells. n=3. C, UMAP plot and keys to the clustering numbers and cell types are shown to the right. D, UMAP plot of sham and punched group. E, UMAP plot of marker genes of MSC, tenocyte and chondrocyte. MSC, mesenchymal stromal cell. Source data are provided as a Source Data file.

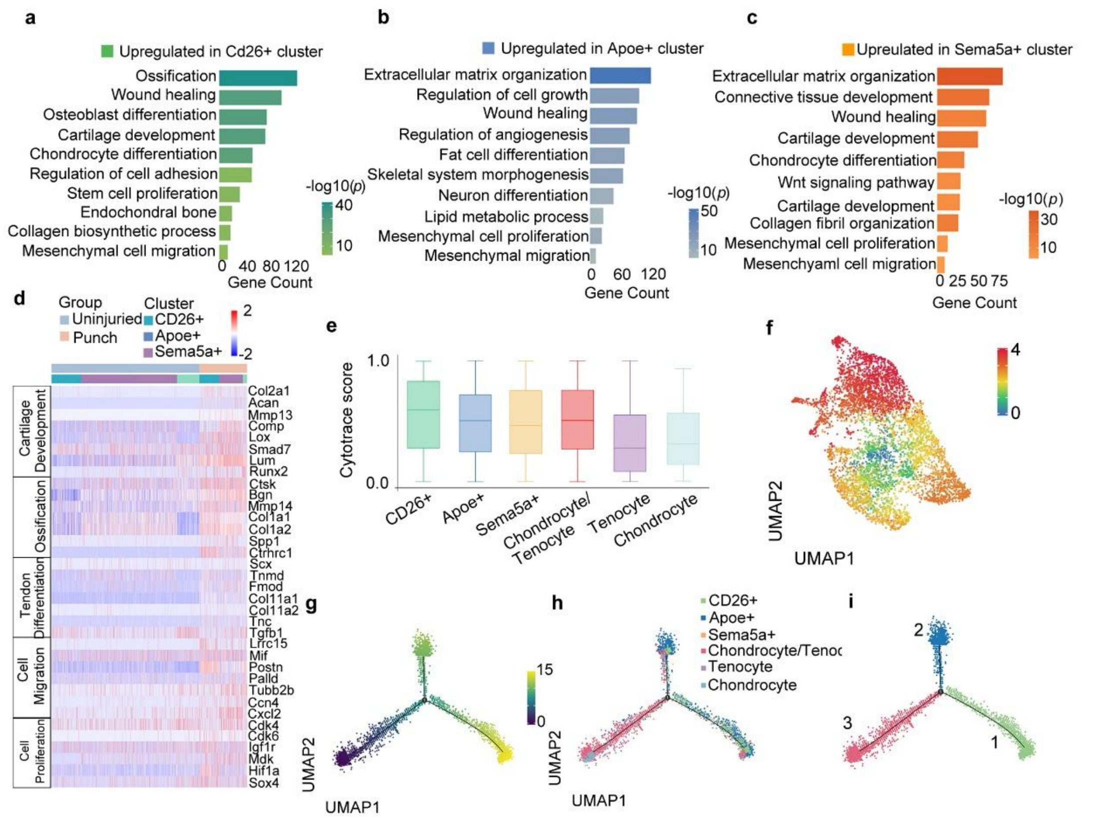

Figure S5 A, GO pathway analysis of upregulated genes in CD26^{+} cluster. B, GO pathway analysis of upregulated genes in Apoe^{+} cluster. C, GO pathway analysis of upregulated genes in Sema5a^{+} cluster. D, Heat map of upregulated genes involved in different biological process in three clusters mentioned above. E, Cytotrace score of clusters CD26^{+} TSPC, Apoe^{+} progenitor, Sema5a^{+} progenitor, chondrocyte/tenocyte, tenocyte and chodnrocyte from uninjured and punched group. F, Pseudotime analysis of CD26^{+} TSPC, Apoe^{+} progenitor, Sema5a^{+} progenitor, chondrocyte/tenocyte, tenocyte and chondrocyte in merged group. G-I, Pseudotime, clusters and states of Pseudotime analysis.

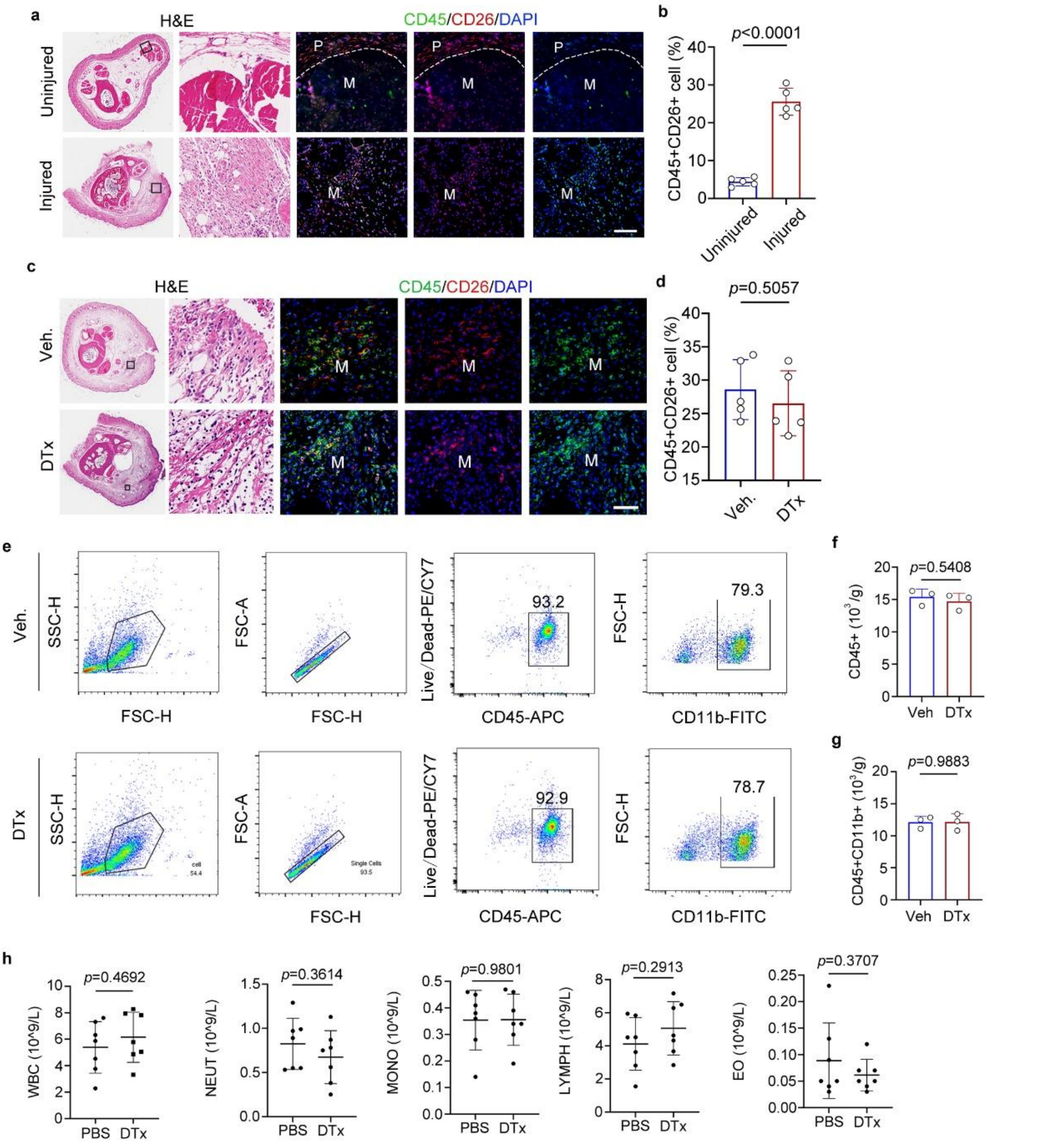

Figure S6. A-B, Immunofluorescent analysis of CD45 and CD26 of uninjured and injured Achilles tendons. n=5. Two-tailed unpaired Student's t test. C-D, Immunofluorescent analysis of CD45 and CD26 of Veh. and DTx group. n=5. Two-tailed unpaired Student's t test. E-G, Flow cytometry analysis of $CD45^+CD11b^+$ myeloid cells in Veh. and DTx group. n=3. Two-tailed unpaired Student's t test. H, Peripheral blood routine examination of PBS and DTx group in CD26-hDTR mice. n=7. Two-tailed unpaired Student's t test. Veh., vehicle, DTx, Diphtheria toxin. Scale bar: 100μm. Data shown are mean ± SEM. Source data are provided as a Source Data file.

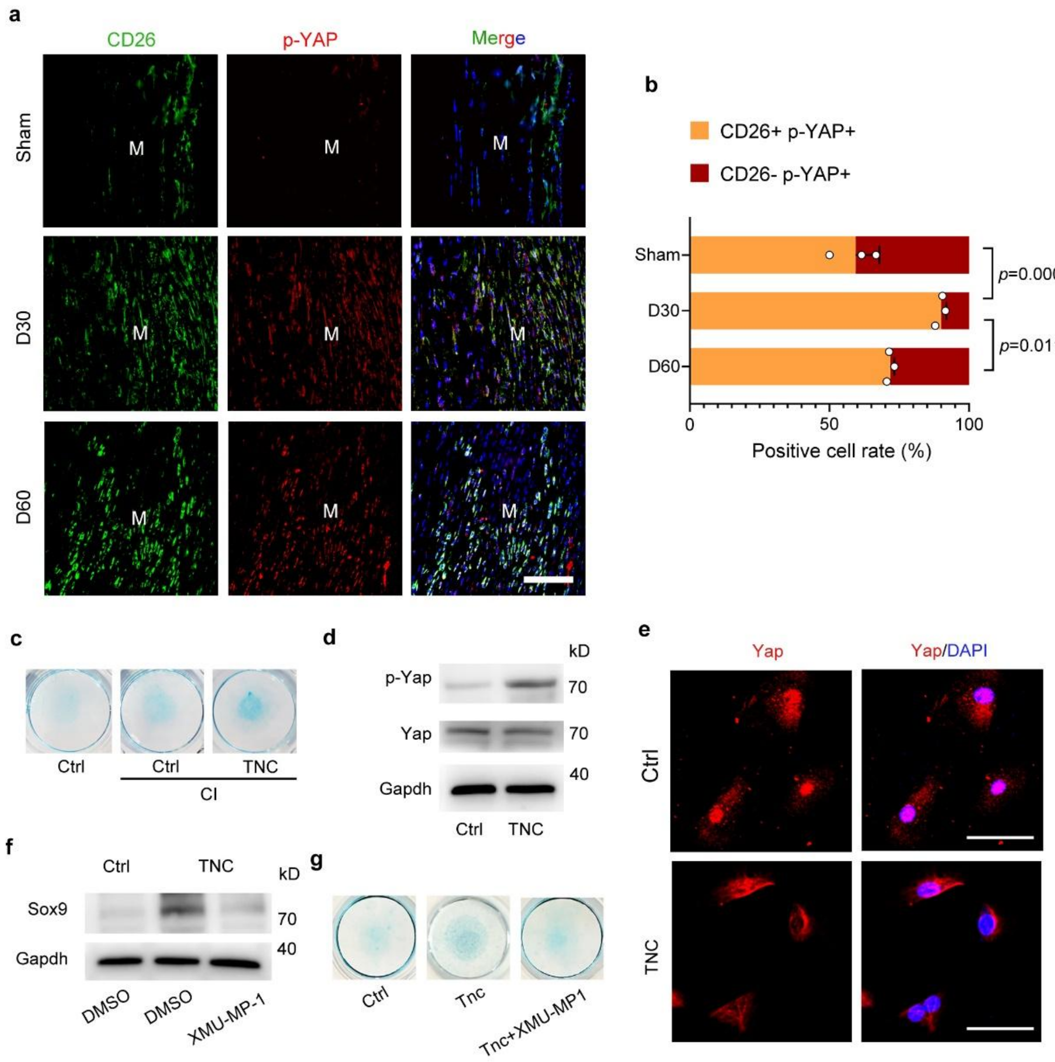

Figure S7 A-B, Immunofluorescent analysis of p-Yap and CD26 in sham, 30 dpi and 60 dpi group. n=3. One way ANOVA with Turkey's test. Scale bar: 100μm. C, Alcian blue staining of CD26$^+$ TSPCs treated with TNC or BSA (bow serum album) control. CI: chondrogenic induction. D, Western blot analysis of phosphorylated-Yap (p-Yap) and Yap in CD26$^+$ TSPCs treated with TNC or BSA control. E, Immunofluorescent analysis of Yap in CD26$^+$ TSPCs treated with TNC or BSA control. F, Western blot analysis of Col2a1 and Sox9 in CD26$^+$ TSPCs treated with dmso or xmu-mp-1 prior to TNC or BSA control stimulation. G, Alcian blue staining of CD26$^+$ TSPCs treated with BSA control, TNC or TNC with XMU-MP-1. Data shown are mean ± SEM. Source data are provided as a Source Data file.

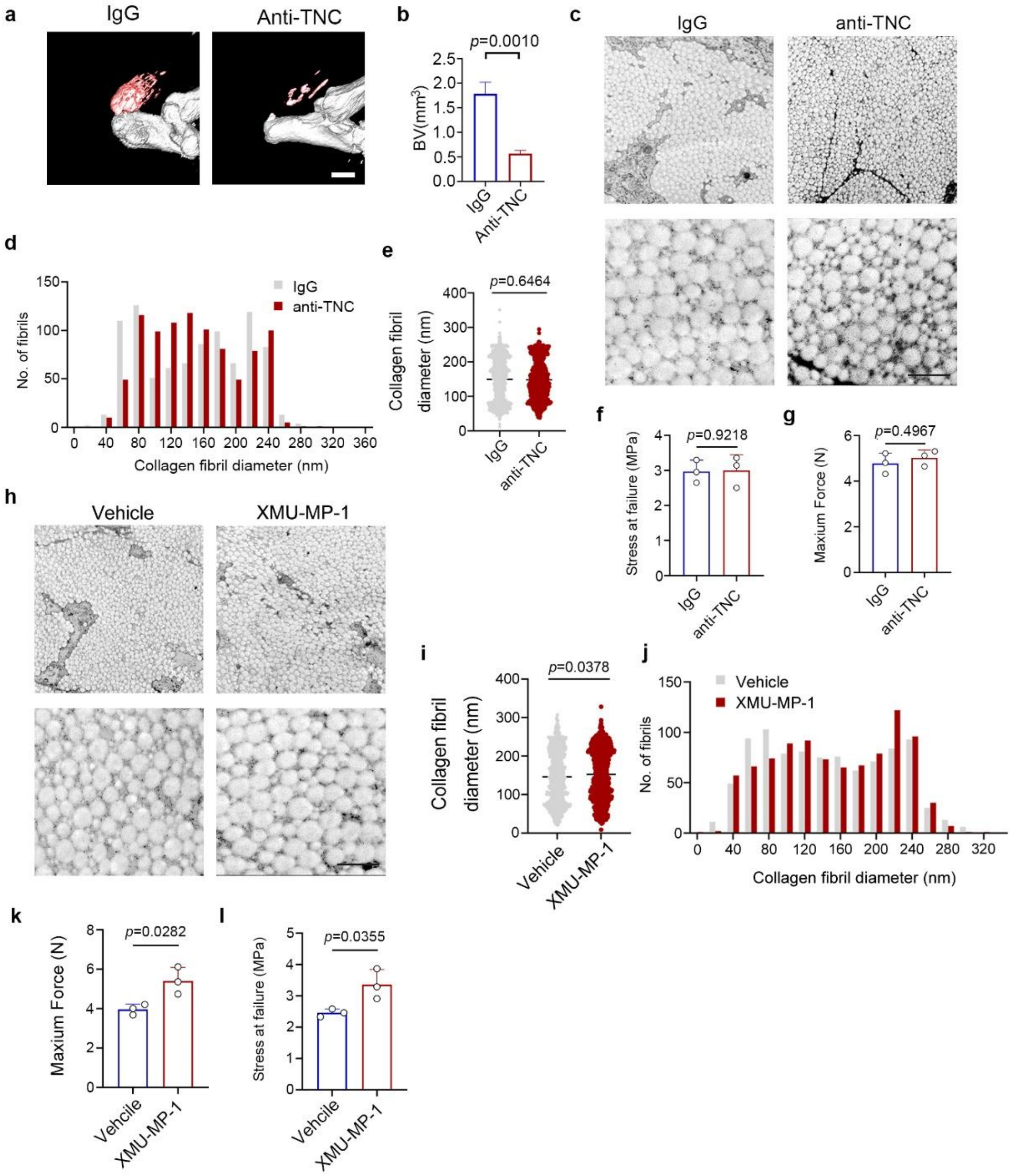

Figure S8. A-B, Micro-CT analysis and quantification of ectopic bone in IgG and anti-TNC group. C, TEM images of cross section of Achilles tendons from IgG and anti-TNC antibody group. Scale bar: 500nm. D-E, Bar graph and dot plot presents the aggregated distribution of collagen fibril diameters using high-magnification TEM images from n = 3 mice per group. Two-tailed unpaired Student's t test. F-G, Quantification of Maxium Force and Stress at failure of tendons from injured with IgG treatment or anti-TNC antibody treatment. n=3. Two-tailed unpaired Student's t test. H, TEM images of cross section of Achilles tendons from vehicle and XMU-MP-1 group. Scale bar: 500nm. I-J, Bar graph and dot plot presents the aggregated distribution of collagen fibril diameters using high-magnification TEM images from n = 3 mice per group. Two-tailed unpaired Student's t test. K-L,

Quantification of Maxium Force and Stress at failure of tendons from vehicle and XMU-MP-1 group. n=3. Two-tailed unpaired Student’s t test. CI: chondrogenic induction. Data shown are mean ± SEM. Source data are provided as a Source Data file.
