# Supplementary material for: A CD26+ tendon stem progenitor cell population contributes to tendon repair and heterotopic ossification
Source: Nat Commun. 2025 Jan 16;16:749. doi: 10.1038/s41467-025-56112-5 (PMC11739514; doi:10.1038/s41467-025-56112-5)
Supplement: Supplementary file 2 — Reporting Summary [file 41467_2025_56112_MOESM2_ESM.pdf]

nature portfolio

Corresponding author(s): Hui Liu

Last updated by author(s): 2024/10/13

Reporting Summary

Nature Portfolio wishes to improve the reproducibility of the work that we publish. This form provides structure for consistency and transparency in reporting. For further information on Nature Portfolio policies, see our Editorial Policies and the Editorial Policy Checklist.

Statistics

For all statistical analyses, confirm that the following items are present in the figure legend, table legend, main text, or Methods section.

n/a	Confirmed	
☐	☒	The exact sample size (*n*) for each experimental group/condition, given as a discrete number and unit of measurement
☐	☒	A statement on whether measurements were taken from distinct samples or whether the same sample was measured repeatedly
☐	☒	The statistical test(s) used AND whether they are one- or two-sided<br>*Only common tests should be described solely by name; describe more complex techniques in the Methods section.*
☒	☐	A description of all covariates tested
☒	☐	A description of any assumptions or corrections, such as tests of normality and adjustment for multiple comparisons
☐	☒	A full description of the statistical parameters including central tendency (e.g. means) or other basic estimates (e.g. regression coefficient) AND variation (e.g. standard deviation) or associated estimates of uncertainty (e.g. confidence intervals)
☐	☒	For null hypothesis testing, the test statistic (e.g. *F*, *t*, *r*) with confidence intervals, effect sizes, degrees of freedom and *P* value noted<br>*Give P values as exact values whenever suitable.*
☒	☐	For Bayesian analysis, information on the choice of priors and Markov chain Monte Carlo settings
☒	☐	For hierarchical and complex designs, identification of the appropriate level for tests and full reporting of outcomes
☒	☐	Estimates of effect sizes (e.g. Cohen's *d*, Pearson's *r*), indicating how they were calculated

Our web collection on statistics for biologists contains articles on many of the points above.

Software and code

Policy information about availability of computer code

Data collection	Oympus/IX83, 10X Genomics (Cell Ranger), FACSAriaII BD, Leica LAX
Data analysis	Data were analyzed with Flowjo, v10 image J v1.54k, R v 4.2.2, GraphPad Prism 7.

For manuscripts utilizing custom algorithms or software that are central to the research but not yet described in published literature, software must be made available to editors and reviewers. We strongly encourage code deposition in a community repository (e.g. GitHub). See the Nature Portfolio guidelines for submitting code & software for further information.

Data

Policy information about availability of data

All manuscripts must include a data availability statement. This statement should provide the following information, where applicable:

- Accession codes, unique identifiers, or web links for publicly available datasets
- A description of any restrictions on data availability
- For clinical datasets or third party data, please ensure that the statement adheres to our policy

The murine ScRNA-seq and RNA-seq generated in this study have been deposited in GSA database under accession code [CRA020509]. T?he human ScRNA-seq generated in this study have been deposited in GSA database under accession code [HRA009395]. Source data are provided with this paper.

Research involving human participants, their data, or biological material

Policy information about studies with human participants or human data. See also policy information about sex, gender (identity/presentation), and sexual orientation and race, ethnicity and racism.

Reporting on sex and gender	The patients were as follows: mean age (20.3), gender (33.3% female,66.6% male). Sex or gender was not considered in the study design. Sex and/or gender of participants was determined based on their identification cards provided by the goverment.
Reporting on race, ethnicity, or other socially relevant groupings	No socially relevant categorization variable was used in the current study.
Population characteristics	For human spinal and tendon tissues collection, unaffected Achilles tendons from amputated limbs from 3 patients with osteosarcoma (2male and 1 female, average age: 20 years old) and spinal ligament tissues from 3 patients 2 male and 1 female, average age: 20.7 years old who fulfilled the criteria for correction of scoliosis were collected.
Recruitment	For human spinal and tendon tissues collection, aged-matched patients who fullfilld the criteria with with amputation surgery or correction of scoliosis were included. All tendon and ligament tissues used were based on their availability, which would not affect our conclusion.
Ethics oversight	All procedures were approved by the Medical Ethics Committee of First Affiliated Hospital of Sun Yat-sen University.

Note that full information on the approval of the study protocol must also be provided in the manuscript.

Field-specific reporting

Please select the one below that is the best fit for your research. If you are not sure, read the appropriate sections before making your selection.

☒ Life sciences ☐ Behavioural & social sciences ☐ Ecological, evolutionary & environmental sciences

For a reference copy of the document with all sections, see nature.com/documents/nr-reporting-summary-flat.pdf

Life sciences study design

All studies must disclose on these points even when the disclosure is negative.

Sample size	No statistical method was used to pre-determine sample size and sample size was determined based on previous similarly designed experiments.
Data exclusions	No data was excluded from analysis.
Replication	The experimental findings were reproducible and were replicated at least for three times.
Randomization	Each sample is marked with a different number before grouping and then grouped by a random grouping procedure..
Blinding	The experiment operator is different from the experiment designer, and the operator does not know the grouping situation.

Reporting for specific materials, systems and methods

We require information from authors about some types of materials, experimental systems and methods used in many studies. Here, indicate whether each material, system or method listed is relevant to your study. If you are not sure if a list item applies to your research, read the appropriate section before selecting a response.

Materials & experimental systems

n/a	Involved in the study	
☐	☒	Antibodies
☒	☐	Eukaryotic cell lines
☒	☐	Palaeontology and archaeology
☐	☒	Animals and other organisms
☒	☐	Clinical data
☒	☐	Dual use research of concern
☒	☐	Plants

Methods

n/a	Involved in the study	
☒	☐	ChIP-seq
☐	☒	Flow cytometry
☒	☐	MRI-based neuroimaging

Antibodies

Antibodies used

Histology:
anti-Scx antibody (Abcam, 1:100, CAT: ab58655); anti-Col1a1 antibody (Abcam, 1:100, CAT: ab270993); anti-Tnc antibody (Abcam, 1:100, CAT: ab108930); anti-Fmod antibody (Proteintech, 1:100, CAT: 60108-1-lg); anti-Sox9 antibody (Abcam, 1:100, CAT: ab185230); anti-Sp7 antibody (Abcam, 1:100, CAT: ab209484); anti-colagen II antibody (Abcam, 1:100, CAT: ab34712); anti-Ocn antibody (Abcam, 1:100, CAT: ab93876); anti-Dpp4 antibody (Abcam, 1:100, CAT: ab187048); anti-p-Yap1 antibody (Abcam, 1:100, CAT: ab76252). The next day, the sections were washed off with PBS and the slides were probed with HRP secondary antibody (1:200)

Flow cytometry: CD26 (Dpp4)-PE (Bioledgend, 1:100, CAT: 137803); CD90-PE/CY7 (Bioledgend, 1:100, CAT: 140310); CD90-FITC (Biolegend, 1:100, CAT: 140303); CD31-PB450 (Bioledgend, 1:100, CAT: 102421); CD45-APC (Bioledgend, 1:100,CAT:157606); CD45-PB450 (Bioledgend, 1:100, CAT: 103125); CD44-FITC (Bioledgend, 1:100, CAT: 103021); CD11b-FITC (Biolegend, 1:100, CAT: 101205)Ly6a-PE/CY7 (Bioledgend, 1:100, CAT: 108113); CD105-488 (eBioscience, 1:100, CAT: 53-1051-82); CD200-PE/CY7 ((Bioledgend, 1:100, CAT: 123818); anti-leptin receptor (Abcam, 1:100, CAT: ab216690); anti-Tppp3 (Abcepta, 1:100, CAT: AP5004b); anti-Apoe (Abcam, 1:100, ab183597); anti-Sema5a (R&D system, 1:100, CAT: AF5896-SP)

Validation

All of the antibodies were validated by the suppliers as indicated in quality assurance.

Animals and other research organisms

Policy information about studies involving animals; ARRIVE guidelines recommended for reporting animal research, and Sex and Gender in Research

Laboratory animals

Mouse strains: c57BL/6J, CD26-cerERT2-hDTR, mZsGmT, TNC-/- c57BL/6J mice were purchased from the GemPharmatech and Cyagen All mice were aged matched at 9 weeks when experimenr were performed.

Wild animals

This study did not include wild animals.

Reporting on sex

Female(50%) , Male(50%) . This study did not consider sex in study design.

Field-collected samples

This study did not include samples collected from the field.

Ethics oversight

All procedures were approved by Institutional Animal Care and Use Committee, Sun yat-sen University.

Note that full information on the approval of the study protocol must also be provided in the manuscript.

Plants

Seed stocks

Report on the source of all seed stocks or other plant material used. If applicable, state the seed stock centre and catalogue number. If plant specimens were collected from the field, describe the collection location, date and sampling procedures.

Novel plant genotypes

Describe the methods by which all novel plant genotypes were produced. This includes those generated by transgenic approaches, gene editing, chemical/radiation-based mutagenesis and hybridization. For transgenic lines, describe the transformation method, the number of independent lines analyzed and the generation upon which experiments were performed. For gene-edited lines, describe the editor used, the endogenous sequence targeted for editing, the targeting guide RNA sequence (if applicable) and how the editor was applied.

Authentication

Describe any authentication procedures for each seed stock used or novel genotype generated. Describe any experiments used to assess the effect of a mutation and, where applicable, how potential secondary effects (e.g. second site T-DNA insertions, mosiacism, off-target gene editing) were examined.

Flow Cytometry

Plots

Confirm that:

☒ The axis labels state the marker and fluorochrome used (e.g. CD4-FITC).

☒ The axis scales are clearly visible. Include numbers along axes only for bottom left plot of group (a 'group' is an analysis of identical markers).

☒ All plots are contour plots with outliers or pseudocolor plots.

☒ A numerical value for number of cells or percentage (with statistics) is provided.

Methodology

Sample preparation

Cells were isolated from mouse tendon and ligament tissues as described previously. Then they were digested in a mix of

Sample preparation	collagenase II (Roche, 3 mg/ml) and dispase II (Sigma-Aldrich, 4 mg/ml), prepared in DMEM for 1 hour at 37 °C. Digestions were subsequently quenched with 10% FBS DMEM and filtered through 40μm sterile strainers. Cells were then washed in PBS with 0.04% BSA, counted and resuspended. Then cells were stained with antibodies and analyzed by flow cytometry or used for FACS.
Instrument	FACSAriaII BD, FACS Symphony BD
Software	Flowjo v10
Cell population abundance	A small fraction of the sorted cells were run on the Aria II, then experiments were performed using the same gating strategy.
Gating strategy	1)Mouse CD26+ TSPCs and Sema5a+ progenitors: FSC-A/SSC-A(cells)→FSC-H/FSC-A(singlets)→LiveDead/(CD45-CD31-)→Sca1+/FSC-A→Sema5a+CD26-, Sema5a-CD26+<br>2) Human CD26+ MSCs: FSC-A/SSC-A(cells)→FSC-H/FSC-A(singlets)→LiveDead/(CD45-CD31-)→CD26+/FSC-A<br>3) Stem cell marker detection for mouse CD26+ TSPCs :FSC-A/SSC-A(cells)→FSC-H/FSC-A(singlets)→LiveDead/(CD45-CD31-)→Sca1+/FSC-A→Sema5a-CD26+→CD44, CD90, CD105, Scx, Lepr<br>4) Stem cell marker detection for human CD26+ TSPCs: FSC-A/SSC-A(cells)→FSC-H/FSC-A(singlets)→LiveDead/(CD45-CD31-)→CD26+/FSC-A→CD44, CD105, CD90, Lepr<br>4) Tppp3 dection in mouse CD26+ TSPCs, Apoe+ progenitors and Sema5a+ progenitors: FSC-A/SSC-A(cells)→FSC-H/FSC-A(singlets)→LiveDead/(CD45-CD31-)→Sca1+/FSC-A→Sema5a-CD26+, Sema5a+CD26-, Sema5a-CD26-→Tppp3/FSC-A<br>5) myeloid cell detection: FSC-H/SSC-H(cells)→FSC-H/FSC-A(singlets)→LiveDead/CD45→FSC-H/CD11b

☒ Tick this box to confirm that a figure exemplifying the gating strategy is provided in the Supplementary Information.
